# Impact of the COVID-19 pandemic in the Portuguese population: Consumption of alcohol, stimulant drinks, illegal substances, and pharmaceuticals

**DOI:** 10.1371/journal.pone.0260322

**Published:** 2021-11-19

**Authors:** Sara Fernandes, Milaydis Sosa-Napolskij, Graça Lobo, Isabel Silva

**Affiliations:** 1 School of Medicine and Biomedical Sciences of the University of Porto (ICBAS-UP), Porto, Portugal; 2 CINTESIS–Center for Health Technology and Services Research, Porto, Portugal; 3 FMUP–Faculty of Medicine of the University of Porto, Porto, Portugal; 4 Laboratory of Pharmacology and Neurobiology–Department of Immuno-physiology and Pharmacology, School of Medicine and Biomedical Sciences (ICBAS-UP), Porto, Portugal; 5 Center for Drug Discovery and Innovative Medicines (MedInUP), School of Medicine and Biomedical Sciences (ICBAS-UP), Porto, Portugal; 6 Molecular Neurobiology Group–Institute for Molecular and Cell Biology (IBMC), Institute for Research and Innovation in Health (i3S), Porto, Portugal; 7 Department of Neurology–Centro Hospitalar Universitário do Porto (CHUPorto), Porto, Portugal; Konkuk University, REPUBLIC OF KOREA

## Abstract

**Background:**

The measures implemented by governments worldwide to control and prevent the spread of the COVID-19 have impacted the populations and directly influenced individuals’ quality of life and consumption habits.

**Objective:**

This work investigates the Portuguese population’s changes in alcohol, stimulants drinks, illegal substances, and pharmaceutical consumptions habits during the COVID-19 pandemic.

**Methods:**

An online questionnaire comprising seven groups of questions–with one group referring to alcohol, stimulant drinks, illegal substances, and pharmaceuticals consumption habits–was made available to the general adult population of mainland Portugal from the 26^th^ January through the 31^st^ of March 2021. After applying the inclusion criteria, 1666 questionnaires were selected and analysed using descriptive and inferential statistics.

**Results:**

Our results show that 48.9% of the participants have alcohol drinking habits and increased their alcohol consumption by 16% after the beginning of the COVID-19 pandemic lockdown. Furthermore, 8.7% of the respondents felt the need to increase their consumption of stimulant drinks, especially coffee, the most consumed stimulant drink (77.9%). We also observed that of the 3.1% of respondents who are usual consumers of illegal substances, 26.9% increased their consumption of these substances during the COVID-19 pandemic. Concerning pharmaceuticals, 23.2% of the respondents expressed their need to take a therapeutic drug after the start of the COVID-19 pandemic. The profile of common consumers of alcohol, stimulant drinks, illegal substances, and pharmaceuticals in the COVID-19 pandemic context is contrasting and varies according to gender, age, and employment status.

**Conclusions:**

The COVID-19 pandemic led to an increase in the consumption of alcohol, stimulant drinks, illegal substances, and pharmaceuticals prescribed to treat anxiety, depression, and sleep changes in the Portuguese population. These new consumption patterns have probably aggravated domestic violence, mental diseases, and impairment of family quality of life in the Portuguese population.

## Introduction

The Coronavirus disease 2019 (COVID-19) caused by the Severe Acute Respiratory Syndrome coronavirus 2 (SARS-CoV-2) has caused more than 200 million cases and led to over 4 million deaths worldwide since it was first reported in Wuhan, China, on the 31^st^ of December 2019 [1, 2]. Upon the declaration of a pandemic outbreak on the 11^th^ of March 2020 [3], the urgency to control and prevent the spread of the COVID-19 forced governments around the world to implement rules and restrictions that have had unavoidable negative social, economic, and health consequences widely reported [4–8]. These restrictions have impacted, for example, populations’ food habits, ultimately altering the gut microbiota, which influences general health. Recently, it has been suggested that the influence of food in the gut microbiota and the axis brain-gut homeostasis may contribute to the susceptibility and clinical outcome of the COVID-19 [9].

In Portugal, the first case of COVID-19 was diagnosed on the 2^nd^ of March, 2020 [10]. By the 16^th^ of March 2020, 1.111 cases had been confirmed, and the first 12 deaths due to the COVID-19 had been registered, leading to the declaration of the first national state of emergency on the 18^th^ of March and the subsequent implementation of emergency measures such as the mandatory lockdown and stay-at-home rules [11]. Since then, and though under control, the pandemic has persisted, forcing authorities to maintain strict sanitary measures that caused an enormous impact on the Portuguese population with direct incidence on the mental health of individuals [12–14].

Reports on the COVID-19 pandemic mental health consequences in the Portuguese population have described moderate to severe levels of psychological impact concerning anxiety [13, 15, 16]. Also, a study analysing the impact of the COVID-19 in Portugal, Brazil, and Colombia has uncovered vulnerabilities regarding poor sleep quality and mild to moderate suicidal ideation in the population of those countries [15]. Moreover, moderate levels of fear of the COVID-19 have been reported in the Portuguese population [17], and high levels of depressive symptoms, stress, and anxiety have also been evidenced among health professionals [18].

At least one study has addressed the association of psychological factors with the consumption of wine in the COVID-19 context in Portugal and Spain and has reported reasons of pleasure (e.g., experiencing a specific wine or relaxing) rather than negative feelings related to the COVID-19 to drink wine [19]. Alcohol consumption has been associated with the COVID-19 severity and other comorbidities [20] and problems such as domestic violence during the COVID-19 pandemic [21].

However, to the best of our knowledge, studies on the consumption of alcohol, stimulant drinks, pharmaceuticals, and drugs concerning the COVID-19 are scarce.

In this paper, we investigate the impact of the COVID-19 in the Portuguese population concerning the consumption of alcohol, stimulant drinks, illegal substances, and pharmaceuticals.

## Data and methods

### Study design

We carried out a cross-sectional exploratory-descriptive study using an online questionnaire targeted at the general adult population of mainland Portugal. The survey was available for completion for 65 days, from the 26^th^ of January through the 31^st^ of March, 2021.

The online questionnaire was built in the platforms *Inquéritos UP* (from the University of Porto) and Google Forms. Furthermore, its weblinks were disseminated in various social media (e.g., Facebook, Instagram, and WhatsApp) [22] and e-mailed to members of the academic community of the University of Porto and other Portuguese Universities that agreed to contribute to this project. All the participants were informed that upon submission of the questionnaire and acceptance of the consent statement, their answers would be anonymised using a unique individual identifier. The participants were also informed that their responses would be used in a scientific study conducted in the framework of a Master’s Degree in Forensic Medicine of the School of Medicine and Biomedical Sciences of the University of Porto (ICBAS-UP).

The questionnaire was divided into seven groups of questions comprising sixty-two questions in total. The first group aimed to collect the participants’ demographic data, such as gender, age, marital status, employment status, education degree, region of residence, cohabitation, number of household members, and health conditions. The second group addressed personal situations concerning the COVID-19 infection, financial condition, teleworking, and the psychological impact of the pandemic. The third group focused on the participants’ alcohol, stimulant drinks, illegal substances, and pharmaceuticals consumption habits during the COVID-19 pandemic. The fourth batch of questions requested information about changes in family relationships and domestic violence, while the fifth group focused on issues related to SARS-CoV-2 susceptibility and COVID-19-related health problems. In group six, we requested information about the participants’ perception of rapid tests and COVID-19 vaccination; and group seven aimed at learning the participants’ opinions on the future implications that may arise from the time course of the COVID-19 pandemic and to evaluating their overall satisfaction.

In this paper, we focused on the first and third groups of answers collected with the survey to analyse the demographic characteristics of the participants and their alcohol, stimulant drinks, illegal substances, and therapeutic drugs consumption habits during the COVID-19 pandemic.

The period during which this survey was conducted coincided with the second emergency state decreed by the Portuguese government.

### Inclusion criteria of participants

The participants were required to be over 18 years of age, reside in mainland Portugal, and understand Portuguese to be included in the study. We excluded participants living in the Autonomous Regions of Madeira and Azores because these territories abide by their own health rules and laws which may differ from those implemented in mainland Portugal.

### Data analysis

We used descriptive statistics to examine the participants’ answers using frequencies and percentages, and independent-samples *t-*tests were performed to assess the mean ± SD differences between ages in the groups. The *N* represents the number of individuals. The statistical analysis of the data was carried out using IBM SPSS Statistics for Windows software, Version 27.0 (Armonk, NY: IBM Corp), and graph representations were produced with GraphPad Prism version 8.0.0 for Windows software (La Jolla, USA; RRID: SCR 002798). *P* < 0.05 values were considered statistically significant.

### Sample

**Fig 1:**
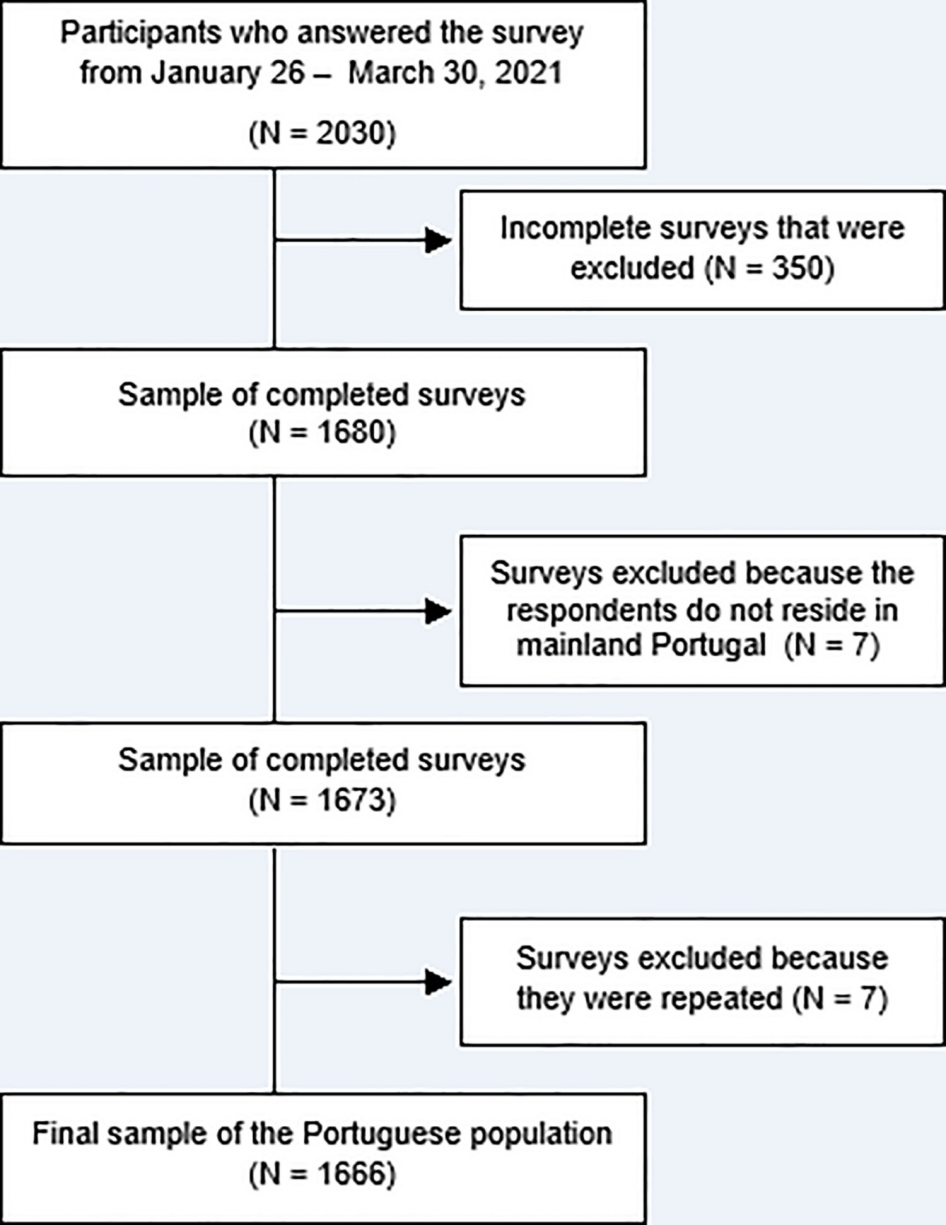
Flowchart of sampling process of the Portuguese population.

We obtained 2030 surveys, of which 350 were incomplete and had to be excluded. Of the 1680 surveys, 14 were also excluded because the participants did not reside in mainland Portugal (N = 7) or had answered the survey twice or more times. Therefore, the final sample included 1666 participants (Fig 1).

### Ethics

This study was approved by the Ethics Committee of the CHUP—University Hospital and the School of Medicine and Biomedical Sciences–ICBAS-UP (References: 2020/CE/P028 and P340/CETI/ICBAS). Likewise, the research project and the questionnaire follow the principles outlined in the Code of Ethics of the World Medical Association (Declaration of Helsinki).

## Results

### Sample characteristics

We surveyed 1666 Portuguese participants. Most of the respondents were women (N = 1219, 73.2%) (Table 1) with a mean age of 32 ± 13 years, while 26.8% of the respondents were men (N = 447), with a mean age of 35 ± 15 years, *p* > 0.05.

**Table 1:** Sociodemographic characteristics of the sample (N = 1666).

		Frequency (N)	%
**Gender**	Female	1219	73.2
	Male	447	26.8
**Age group**	[18–30]	931	55.9
	[31–50]	503	30.2
	[51–65]	197	11.8
	[66–88]	35	2.1
**Marital Status**	Single / divorced / widowed	1134	68.1
	Married / Partnered	532	31.9
**Residence region**	North	1398	83.9
	Centre	193	11.6
	Lisbon and Vale do Tejo	45	2.7
	Alentejo	23	1.4
	Algarve	7	0.4
**Academic degree**	1–9 years	41	2.5
	Secondary / Professional education (10–12)	523	31.4
	Higher education bachelor’s, Master’s or doctoral degree	1102	66.1
**Employment status**	Employed	863	51.8
	Unemployed	23	1.4
	Student	723	43.4
	Working student	39	2.3
	Retired	18	1.1

Note: All results are represented in frequency (N, number of individuals) and percentage (%).

The participants ranged from 18 to 88 years of age and were distributed into four groups according to age: young individuals with ages 18 to 30 (55.9%, N = 931), active professionals, with ages 31 to 50 (30.2%, N = 503), mature and professionally stable adults, with ages 51 to 65 (11.8%, N = 197) and elderly with ages from 66 to 88 (2.1%, N = 35). Most of the participants live in the North, i.e., 83.9%, (N = 1398), and in the Centre, i.e., 11.6% (N = 193) regions of Portugal; 66.1% (N = 1102) have higher education (bachelor, master, or doctoral degree), and 51.8% (N = 863) are employed. Lastly, 31.9% (N = 532) of the respondents are married or in a relationship, while 68.1% (N = 1134) are single, divorced, or widowed (Table 1).

### Alcohol and stimulant drinks

**Fig 2:**
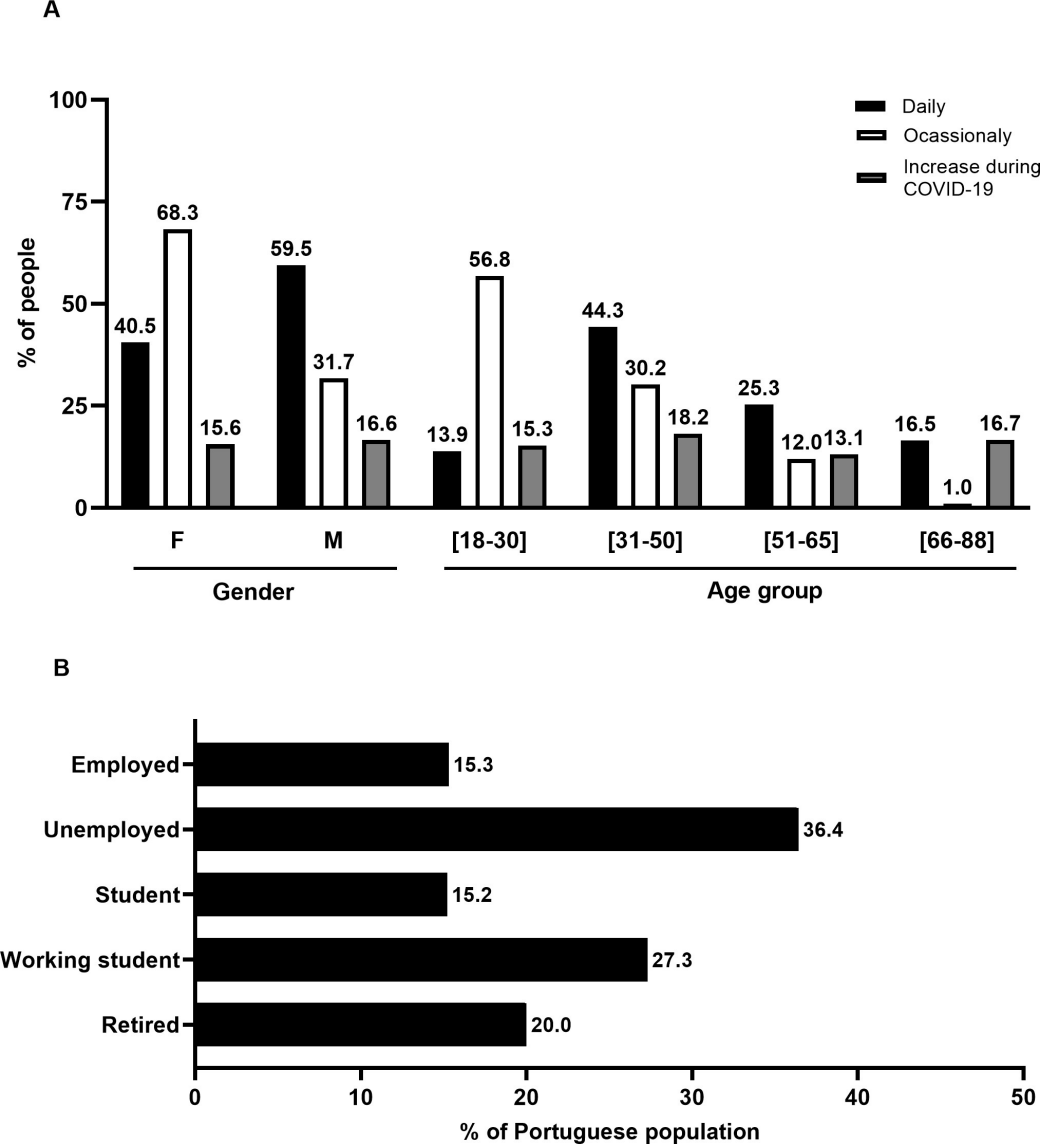
**A.** Consumption habits of alcohol in the Portuguese population. (A) Consumption of alcohol in the sample; the black bars correspond to the daily consumption while the white bars correspond to occasional consumption; the grey bars refer to the increase of alcohol consumption during the COVID-19 pandemic. The Y axis represents the percentage (%) of people; the individuals’ values are above each bar. The X axis represents the groups’ analysis (gender: female (F) and male (M) and age group). **B.** Consumption habits of alcohol in the Portuguese population. (B) Characterisation of people that increased their consumption of alcohol by employment status. The Y axis represents employment status, and the X axis represents the percentage (%) of people that increased their alcohol consumption.

To the questions about their alcohol habits, 48.9% (N = 815) of the participants confirmed that they drink alcohol, while the majority, i.e., 51.1% (N = 851), referred that they do not drink alcohol. Of those who drink alcohol, 44.2% (N = 736) drink only occasionally (age 32 ± 13 years), while 4.7% (N = 79) drink daily (age 49 ± 15 years, *p<0*.*05*). Fig 2A shows the characteristics of the individuals drinking alcohol daily and from time to time, grouped by gender and age. As it can be seen, occasional alcohol consumers are mostly women (68.3%), whereas daily consumers are predominantly men (59.5%).

Most of the occasional alcohol consumers are young people (18 to 30 years old), i.e., 56.8%, while those drinking alcohol daily are professionally active individuals (31 to 50 years old), i.e., 44.3%. In our sample, alcohol consumption increased 16% during the COVID-19 pandemic. As it is shown in Fig 2A, alcohol consumption increased in all age groups, with the groups of professionally active individuals (31 to 50 years old) and male individuals showing the greatest increases, i.e., 18.2% and 16.6%, respectively. Fig 2B represents the increase of alcohol consumption by employment status.

The greatest increase occurred in unemployed individuals, i.e., 36.4%, followed by working students (27.3%).

**Fig 3:**
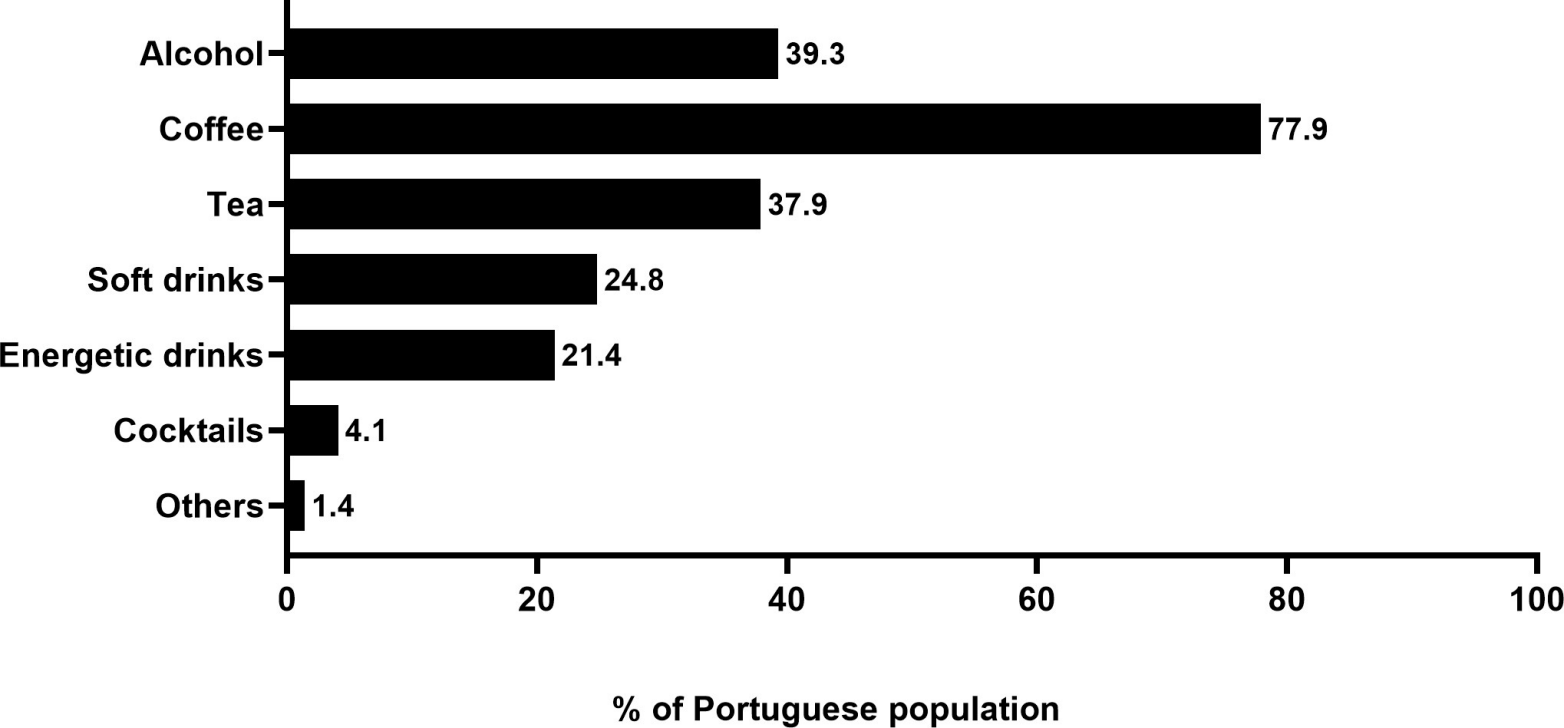
Representation of stimulant drinks that the Portuguese population consumed during the COVID-19 pandemic. The types of stimulant drinks are represented in the Y axis. The percentage (%) of the Portuguese population that increased stimulant drinks consumption is represented in the X axis. The individual value in percentage for each drink is shown in front of each bar.

Concerning stimulant drinks, 8.7% of the individuals felt the need to increase the consumption of these drinks after the beginning of the COVID-19 pandemic. When we examined what stimulant drinks were being consumed, the majority indicated coffee (77.9%), followed by alcohol (39.3%) and tea (37.9%); cocktails (4.1%) were the less consumed drinks (Fig 3).

Both men and women have similar percentages of increase of stimulant drinks consumption, i.e., 8.3% and 8.9%, respectively. The age group with the sharper increase of stimulant drinks consumption was that of young people (9.2%), followed by the group of individuals who are 31–50 years old (8.3%) and those who are 51–65 years old (8.1%). The elderly (66–88 years old) had the smallest increase in stimulant drinks consumption (2.9%).

### Illegal substances

**Fig 4:**
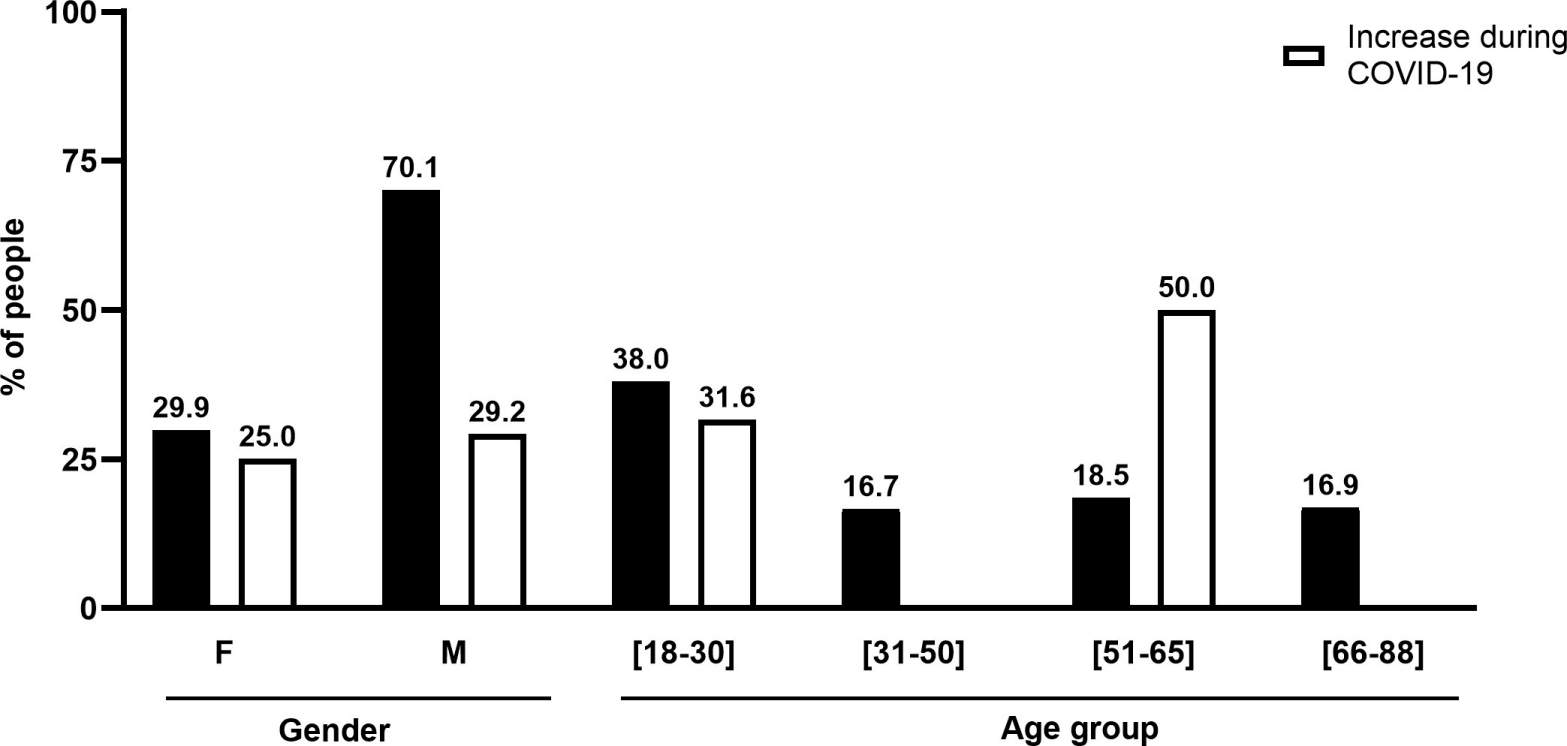
Characterisation of the Portuguese population consuming illegal substances. The black bars correspond to the usual consumption of illegal substances, while the white bars refer to the increase of illegal substances consumption during the COVID-19 pandemic. The Y axis represents the percentage (%) of people; the individual values are above each bar. The analysis by group (gender: female (F) and male (M) and age group) is represented in the X axis.

We could confirm in our sample that 3.1% of the individuals aged 29 ± 13 years are usual consumers of illegal substances. Within that group, 26.9% (age 27 ± 12 years) increased the consumption of illegal substances during the COVID-19 pandemic. Most of the illegal substances consumers are men (70.1%, 34 ± 14 years), mostly young (38.0%, aged 18 to 30 years old) (Fig 4).

However, upon closer examination, we found that the group of individuals who are 51 to 65 years old have the highest increase of illegal substances consumption, i.e., 50.0%, followed by the group of individuals who are 18 to 30 years old (Fig 4). On the other hand, the increase of illegal substances consumption among women and men who are usual consumers of these substances was similar, i.e., 25.0% and 29.2%, respectively (Fig 4). Finally, students and employed individuals also showed an increase in the consumption of illegal substances with 31.3% and 21.1%, respectively.

The analysis of the reasons (Table 2) to increase the use of illegal substances revealed five main motivations: (1) "*I felt alone and/or lonely*," (2) "*I felt anxious and/or stressed*," (3) "*I was unmotivated*, *discouraged or lacking hope*," (4) "*I felt depressed*" and (5) "*I wanted to run away from problems and/or reality*."

**Table 2:** Reasons that promoted the increase of illegal substances consumption during the COVID-19 pandemic.

	Total sample (%)	Gender		Age group		Employment status	
		F (%)	M (%)	[18–30] (%)	[51–65] (%)	Employed (%)	Student (%)
**I felt alone and/or lonely**	87.5	42.9	57.1	100.0	-	14.3	85.7
**I felt anxious and/or stressed**	78.6	54.5	45.5	81.8	18.2	27.3	72.7
**I was unmotivated, discouraged or lacking hope**	90.9	40.0	60.0	80.0	20.0	30.0	70.0
**I felt depressed**	88.9	50.0	50.0	87.5	12.5	25.0	75.0
**I wanted to run away from problems and/or reality**	50.0	66.7	33.3	100.0	-	-	100.0

Note: F: female, M: male. All results are represented in percentage (%).

The main reason for increasing the use of illegal substances was the lack of motivation, encouragement, or hope, indicated by 90.9% of the sample, followed by feelings of depression, mentioned by 88.9% (Table 2). In the analysis of the groups, we observed that most of the women, i.e., 66.7%, age 21 ± 0 years, increased the consumption of illegal substances because they "*wanted to run away from problems and/or reality*," while the main reason invoked by men to increase the use of illegal substances was the feeling "*unmotivated*, *discouraged or lacking hope*," with 60.0%, age 34 ± 15 years.

The group of young individuals (ages 18 to 30) considered that the motivations to increase the consumption of illegal substances were (1) *"feeling alone and/or lonely"* (100%), and (2) *"wanting to run away from problems and/or reality"* (100%). Different reasons were reported by the group of employed individuals, with 30% indicating lack of motivation, encouragement, and hope. Among students, the main reason (100%) for increasing the consumption of illegal substances was *"wanting to run away from problems and/or reality"* (Table 2).

In our questionnaire, the participants could choose more than one reason to use illegal substances. After examining their answers, we found that all the participants chose a combination of options, revealing that the consumption of illegal substances is not motivated by unique or isolated reasons but by a group of causes (S1 Table).

### Pharmaceuticals

The COVID-19 pandemic brought about feelings of stress, anxiety, and even panic among the world population. In Portugal, pharmaceuticals such as antidepressants, sleep inducers, and tranquillizers or anxiolytics are freely and frequently consumed even though they are not sold in pharmacies without prescriptions. Therefore, we considered it pertinent to question our participants about their consumption of pharmaceuticals in this new environment.

**Fig 5:**
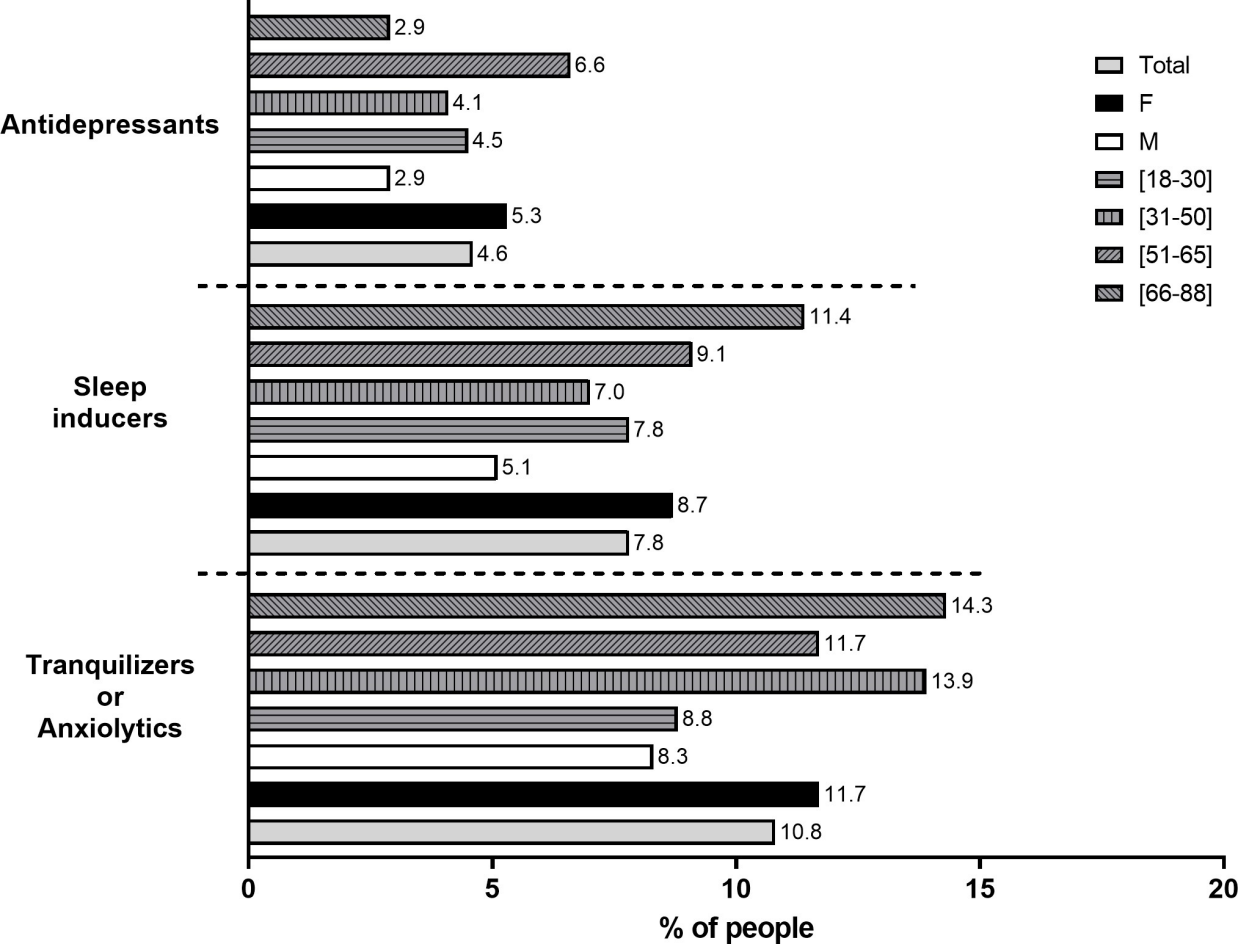
Consumption of pharmaceuticals, namely antidepressants or anxiolytics, sleep inducers and tranquillizers in the Portuguese population. The bars represent the percentage (%) of each analysis group; the grey bars represent a percentage of the total sample, the black bars represent the females (F), the white bars represent the males (M), the grey bars with horizontal stripes represent the individuals aged 18–30 years old, the grey bars with vertical stripes represent the people aged 31–50 years old, the grey bars with left stripes represent the individuals aged 51–65 years old and the grey bars with right stripes represent the individuals aged 66–88 years old. The Y axis represents the types of pharmaceuticals. The X axis represents the percentage (%). Individual values (in percentage) for each group are represented in front of each bar.

During our data analysis, we found high levels of anxiety (data not published) among the participants. We noted that since the start of the COVID-19 pandemic, 23.2% of our respondents needed to take a pharmaceutical. Fig 5 illustrates the increase in the consumption of three groups of pharmaceuticals, with 10.8% for tranquillizers or anxiolytics, 7.8% for sleep inducers, and 4.6% for antidepressants.

In all groups, women revealed the highest increase in pharmaceuticals consumption compared to men. The major consumers of tranquillizers (14.3%) and sleep inducers (11.4%) were the elderly (ages 66 to 88 years old), while the group of individuals aged 51 to 65 was the main consumers of antidepressants (6.6%) (Fig 5).

In response to the question about such pharmaceuticals having been prescribed by a physician, 61.1% of the participants taking tranquillizers or anxiolytics, 44.2% of those taking sleep inducers, and 97.4% of the individuals taking antidepressants answered affirmatively.

Table 3 shows the frequency of consumption of the three groups of pharmaceuticals. As observed, 92.2% of the respondents take antidepressants every day, while 35.6% take tranquillizers with the same frequency. Most of the individuals in our sample take sleep inducers more than once a week (22.5%) or less than once a month (20.2%).

**Table 3:** Frequency of pharmaceuticals consumption.

	Tranquillizers (%)	Sleep inducers (%)	Antidepressants (%)
**Less than once a month**	15.6	20.2	2.6
**Once a month**	8.9	16.2	-
**More than once a month**	19.4	11.6	-
**Once a week**	7.2	10.1	1.3
**More than once a week**	13.3	22.5	3.9
**Every day**	35.6	19.4	92.2

Note: All results are expressed in percentage (%).

The reasons invoked to justify the increase of consumption of these pharmaceuticals were six (Table 4): (1) "*I felt anxious or/and stressed*"; (2) "*I was unmotivated*, *discouraged*, *or lacking hope*"; (3) "*I felt depressed*"; (4) "*I start having sleep changes*"; (5) "*I felt alone and/or lonely*"; (6) "*I wanted to run away from problems and/or reality*."

**Table 4:** Reasons that led to the increase of pharmaceuticals consumption during the COVID-19 pandemic.

	Tranquillizers (%)	Sleep inducers (%)	Antidepressants (%)
**I felt anxious and/or stressed**	87.2	52.7	64.9
**I was unmotivated, discouraged or lacking hope**	22.2	12.4	59.7
**I felt depressed**	17.2	11.6	68.8
**I started having sleep changes**	47.8	82.9	32.5
**I felt alone and/or lonely**	10.6	6.2	22.1
**I wanted to run away from problems and/or reality**	7.2	5.4	10.4

Note: All results are expressed in percentage (%).

The explanations for increasing the consumption of each pharmaceutical diverged. The main motivation for the consumption of tranquillizers was feeling anxiety and/or stress (87.2%), whereas sleep inducers and antidepressants were mostly used to treat sleep changes (82.9%) and feelings of depression (68.8%).

Analysing the same options by gender and age group, we observed that the main motivation for women to increase the use of these therapeutic drugs was sleep changes (84.9%), while in men, the main reason was wanting to run away from problems and/or reality (28.6%). Among young people (ages 18 to 30), the main reason for using pharmaceuticals was feeling unmotivated, discouraged, or lacking hope (61.8%). Active professionals (ages 31 to 50 years) indicated that they used pharmaceuticals mostly to deal with feelings of anxiety and/or stress (33.8%). Mature and professionally stable adults (ages 51 to 65 years old) pointed out that feeling depressed was the main reason for taking pharmaceuticals (14.1%), while the elderly indicated sleep changes as their main motivation to increase the consumption of pharmaceuticals (Table 5).

**Table 5:** Reasons that led to the consumption of pharmaceuticals according to gender and age group.

	Gender		Age group			
	F (%)	M (%)	[18–30] (%)	[31–50] (%)	[51–65] (%)	[66–88] (%)
**I felt anxious and/or stressed**	81.1	18.9	54.9	33.8	9.5	1.8
**I was unmotivated, discouraged or lacking hope**	81.4	18.6	61.8	31.4	5.9	1.0
**I felt depressed**	80.8	19.2	56.6	29.3	14.1	-
**I started having sleep changes**	84.9	15.1	54.6	31.2	11.5	2.8
**I felt alone and/or lonely**	81.8	18.2	56.8	29.5	13.6	-
**I wanted to run away from problems and/or reality**	71.4	28.6	57.1	32.1	10.7	-

Note: All results are expressed in percentage (%).

Concerning the employment status, those with a job indicated that feeling anxious and/or stressed was the main reason to increase their consumption of pharmaceuticals (52.4%), whereas unemployed individuals (3.6%), students (57.1%), and working students (3.6%) identified the desire to run away from problems and/or reality as their main reason to use pharmaceuticals (Table 6).

**Table 6:** Reasons that led to the consumption of illegal substances according to employment status.

	Employed (%)	Unemployed (%)	Student (%)	Working student (%)	Retired (%)
**I felt anxious and/or stressed**	52.4	1.8	43.6	2.2	-
**I was unmotivated, discouraged or lacking hope**	41.2	-	55.9	2.9	-
**I felt depressed**	42.4	1,0	55.6	1.0	-
**I started having sleep changes**	51.8	0.5	45.4	1.8	0.5
**I felt alone and/or lonely**	43.2	-	54.5	2.3	-
**I wanted to run away from problems and/or reality**	35.7	3.6	57.1	3.6	-

Note: All results are expressed in percentage (%).

The use of therapeutic drugs (pharmaceuticals) is not motivated by unique or isolated reasons but by a group of causes (S2 Table).

## Discussion

We aimed to study the influence of the COVID-19 pandemic on alcohol, stimulant drinks, illegal substances, and pharmaceuticals consumption habits of a Portuguese population sample living in mainland Portugal. We used an online survey disseminated in social media and national universities, covering a population aged 18 to 88 years, from all regions of mainland Portugal, all professions, marital status, and academic degrees. Nonetheless, our findings are limited by the relatively low number of elderly participants (ages 66 to 88), representing only 2.1% of the analysed population. This low representation may be a consequence of the means used to disseminate the survey, namely social media, which are not very popular among the elderly. Contacting this population group in person and providing them with support to answer the questionnaire would have contributed to a more balanced number of elderly participants.

The profile of alcohol consumption in our sample (N = 1666) revealed that from the 48.9% of the respondents who drink alcohol, 16% increased their consumption during the COVID-19 pandemic. This increase differs according to gender and age group (Fig 2A); based on our data, we can state that men are the main daily consumers while women drink alcohol occasionally. Young people are the main occasional consumers, whereas individuals aged over 30 are essentially everyday consumers. We observed an increase in alcohol consumption in all age groups, and this increase was similar in women and men but was greater in older people than in young persons. Both the increase in alcohol consumption and alcohol abuse due to the lockdown and social isolation has been reported in the literature [23–29]. Also, the stress and isolation experienced by individuals during the COVID-19 pandemic have been suggested as triggers of alcohol use [30–34]. The emotional state triggered by emergency rules such as lockdown, isolation, and curfew was the most contributing factor to alcohol consumption. The other fact that may contribute to the increase of alcohol consumption is the economic crisis provoked by the COVID-19 pandemic, reflected in the high levels of unemployment that occurred in this period. We observed that the group of unemployed persons showed the highest increase (36.4%, Fig 2B) of alcohol consumption. The increase of alcohol consumption and alcohol abuse during the COVID-19 pandemic, combined with isolation, may cause social consequences and alcohol-related harms [21], increasing domestic violence [35–37], health problems [20], and mental health issues [38, 39].

In our research, we also explored domestic violence associated with alcohol consumption. However, we could not observe a correlation between our sample’s alcohol consumption and domestic violence (unpublished results).

In opposition to what is described by Rehm and collaborators (2020), we did not observe in our group of individuals a reduction in alcohol consumption associated with the COVID-19 pandemic [29]. Instead, we perceived that the lockdowns contributed to an increase of alcohol consumption in accordance with Killgore; the author explored the first six months of the COVID-19 pandemic during the first global emergency state, reporting an increase of alcohol consumption by the population [40]. We applied our survey during the second emergency state, about ten to twelve months after the COVID-19 pandemic was declared by the WHO, and detected an increase in alcohol consumption, mainly in unemployed persons. Similar results are described by other studies showing that the greatest increase in high-risk drinking over the pandemic was also observed predominantly among individuals who were under lockdowns or stay-at-home restrictions [40].

Paralleling the alcohol consumption profile, we also investigated the consumption of stimulant drinks in our sample. We observed that coffee was the main stimulant drink consumed during the COVID-19 pandemic, followed by alcohol and tea (Fig 3). The present results are supported by other authors reporting coffee and other stimulant drinks consumption to cope with the stress generated during the COVID-19-induced lockdown or isolation [41]. Furthermore, the restrictions imposed by governments due to the COVID-19 pandemic have caused major alterations in drinks consumption and eating habits in several countries [42–46].

Here we also highlight the significant increase (26.9%) in the consumption of illegal substances (also designated by addiction substances) observed in our sample. We found that this increase is higher in the group of individuals aged 51 to 65 years old (50%) (Fig 4), as opposed to the group of persons who are 31 to 50 years old and the elderly (ages 66 to 88) group that did not use illegal substances at all. According to recent data, the consumptions of alcohol and illegal substances represent around 1.5% of the global burden of the COVID-19 pandemic in the health systems worldwide and up to 5% in the health systems of some nations [47]. Furthermore, addiction substances may predispose individuals to the COVID-19 infection as they increase the risk for pulmonary infections, especially when associated with (a) pre-existing cardio-pulmonary morbidities, (b) mucociliary dysfunction, (c) compromised immunity, (d) altered health-seeking behaviour, and inadequate access to health care delivery, (e) failure of rehabilitation strategies due to social distancing and (f) housing instability [48].

The motivations to increase the use of illegal substances differ according to the age group. Young people and students mentioned that "*feeling alone and/or lonely*" and "*wanting to run away from problems and/or reality*" were the main motivations to increase their use of addictive substances, whereas the individuals aged 51 to 65 years old, men, and employed, acknowledged that "*feeling unmotivated*, *discouraged*, *or lacking hope*" was the main reason to increase their consumption of illegal drugs (see Table 2). Finally, women justified their increase of illegal substances consumption as a form of "*running away from problems and/or reality*."

Other studies also report that the causes for illegal substances use / abuse are diverse and may be associated with external or internal factors such as (1) epigenetic / genetic / personality traits, (2) family discordance / financial stress / domestic violence / poor inter-personal relationships, (3) underlying psychopathologies: social anxiety, depressive episodes, (4) alleviation of effects of substance withdrawal, (5) seeking to have fun / getting "high," increased stimulant/self-confidence, (6) peer pressure [49–52]. Besides these factors that may have been present in the lockdown during the COVID-19 pandemic, other factors such as job loss may have contributed to the increase of illegal substances consumption [49]. We did not examine what illegal substances were used by the respondents in our sample. However, the results reported in other studies in Europe, analysing cannabis, heroin, cocaine [53], and opioid consumption habits during the COVID-19 pandemic [49, 53], may indicate the specific substances consumed by our respondents.

We also investigated the consumption of pharmaceuticals, particularly antidepressants, sleep inducers, and tranquillizers or anxiolytics. In our sample, 23.2% of the individuals needed to take medication after the beginning of the COVID-19 pandemic. Near half of those persons were taking tranquillizers or anxiolytics (10.8%), and the others were using sleep inducers and antidepressants. Tranquillizers were taken mainly to treat anxiety and stress, which increased during the COVID-19 pandemic in health care workers and other non-healthcare professionals [54]. In our sample, the elderly (ages 66 to 88) were the main consumers of tranquillizers and sleep inducers, which could be related to the COVID-19 stay-at-home and isolation restrictions. Modifications in behaviour and cognition, together with functional decline, have been associated with the COVID-19 stay-at-home recommendation given to older adults [55]. Depression and obsessive-compulsive symptoms (worry about contamination, compulsive handwashing behaviour) [54], post-traumatic stress disorders, and generalised anxiety disorders [56] undermine mental health and contribute to the consumption of antidepressants and sleep inducers. The COVID-19 pandemic triggered several mental health disorders reported in several countries [16, 57–62]. In Portugal, the lockdowns imposed due to the COVID-19 pandemic also aggravated the population’s mental health [63].

We did not explore if our population sample had pre-existing mental health vulnerabilities, though more than 50% of the participants considered themselves healthy (unpublished results). It has been described that people with mental health vulnerabilities may develop a fear of the COVID-19 contamination and consequently experience an increase of anxiety symptoms, developing somatic and obsessive symptoms [64], which may lead to the need to take medication, such as antidepressants. There are reports of young people who have developed anxiety after testing positive for the COVID-19 infection despite their physical symptoms being mild and only needing to quarantine at home [65].

The reasons for taking these pharmaceuticals differ by gender, age group, and employment status. Women referred to "*starting to have sleep changes*" as the main reason for using pharmaceuticals, while men mentioned that "*wanting to run away from problems and/or reality*" was their main motivation for this behaviour. Similarly, those unemployed, students, and working students (Table 6) also indicated "*wanting to run away from problems and/or reality*" as their main motivations for using pharmaceuticals. Finally, the group of individuals aged 31 to 50 years old (Table 5) said that "*feeling anxious and/or stressed*" was their main reason for using pharmaceuticals. Overall, the respondents indicated more than one motivation for using antidepressants, sleep inducers, or tranquillizers (S2 Table). Likewise, the use of illegal drugs was justified by more than one reason.

This work demonstrated that societies are not prepared to face pandemic situations. The rules imposed by government, such as lockdowns, may increase alcohol, stimulant drinks, illegal substances, and pharmaceuticals consumption. Governments should plan to respond to pandemics in a way that the impact on people’s lives is the smallest possible. On the other hand, the mind of the people should be trained in situations of pandemics and lockdown. The families have to be prepared to spend more time together. The dialogue between young people should be stimulated, and more support networks should be created to provide help.

## Conclusions

We show for the first time the impact of the COVID-19 pandemic in the Portuguese population concerning the consumption of four substances groups: alcohol, stimulant drinks, illegal substances, and pharmaceuticals.

Despite the limitations of this work, we observed that the COVID-19 pandemic led to an increase in the consumption of alcohol, illegal substances, and pharmaceuticals prescribed to treat anxiety, depression, and sleep changes in the Portuguese population.

The highest and most worrying increase observed regards the overall addiction to substances, despite most of these substances being pharmaceuticals prescribed by health professionals.

Further examination of domestic violence and anxiety patterns in the Portuguese population and their relationship with alcohol, illegal substances, and pharmaceuticals consumption would be of great relevance to the study of the impact of the COVID-19 in Portuguese society.

## Supporting information

S1 Table Combination of the different reasons that led to increasing the consumption of illegal substances. Note: All results are expressed in percentage (%).
(TIF)

S2 Table Combination of the different reasons that led to increasing the consumption of pharmaceuticals. Note: All results are expressed in percentage (%).
(TIF)
