# Supplementary figures and images for: Impact of the COVID-19 pandemic in the Portuguese population: Consumption of alcohol, stimulant drinks, illegal substances, and pharmaceuticals
Source: PLoS One. 2021 Nov 19;16(11):e0260322. doi: 10.1371/journal.pone.0260322 (PMC8604309; doi:10.1371/journal.pone.0260322)

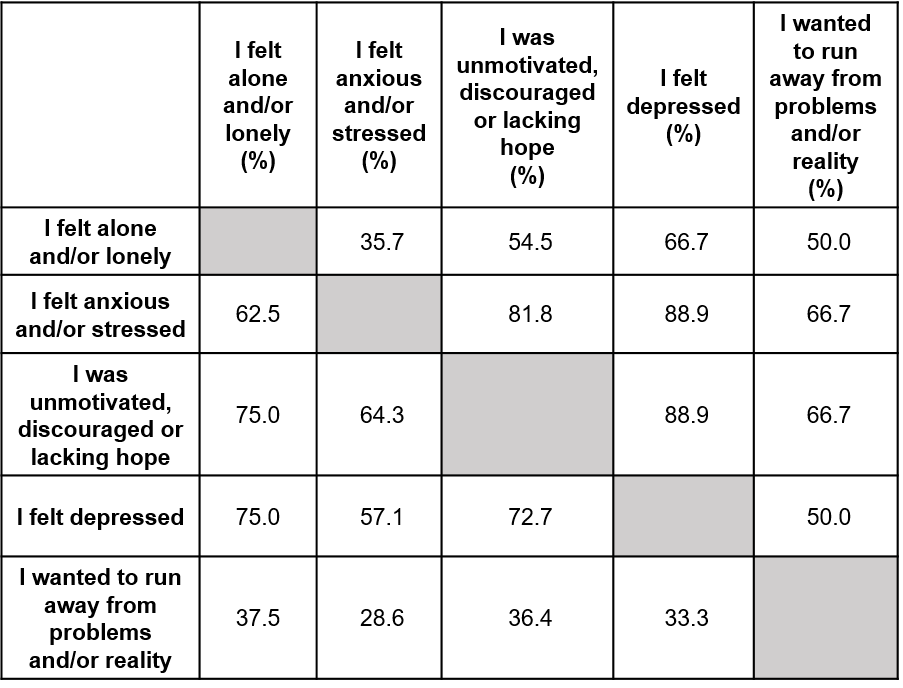

Supplement: S1 Table — Note: All results are expressed in percentage (%). (TIF) [file pone.0260322.s001.tif]

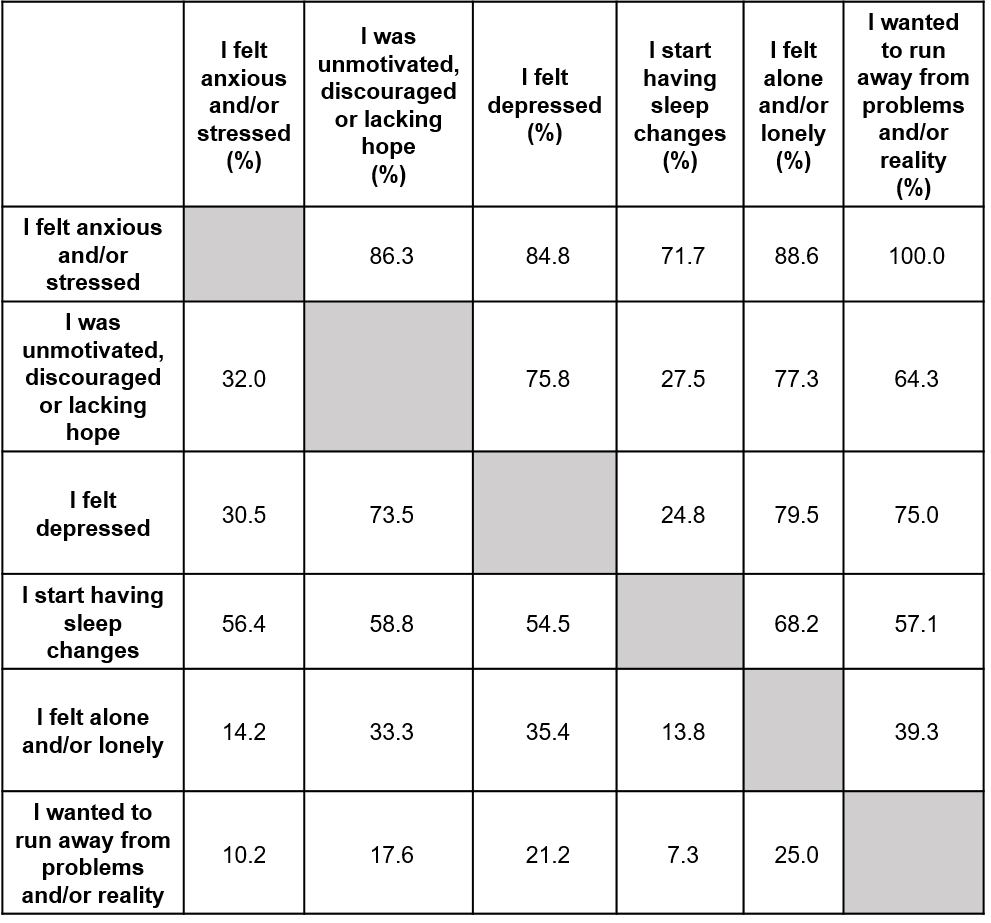

Supplement: S2 Table — Note: All results are expressed in percentage (%). (TIF) [file pone.0260322.s002.tif]
